# Ethical issues related to restrictive practices for anorexia nervosa in young people

**DOI:** 10.1186/s40337-026-01715-6

**Published:** 2026-08-03

**Authors:** Angela Yan, Michele Yeo, Emily Wilson, Jenny O’Neill, Lynn Gillam

**Affiliations:** 1 University of Melbourne, Melbourne, Australia; 2 Murdoch Children’s Research Institute, Melbourne, Australia; 3 The Royal Children’s Hospital, Melbourne, Australia

**Keywords:** Anorexia nervosa, Adolescents, Coercion, Involuntary treatment, Restrictive practices, Restraint, Ethics

## Abstract

**Background:**

Anorexia nervosa (AN) is an eating disorder affecting many young people. In severe AN, complications associated with profound weight loss and malnutrition can arise, and restrictive measures (e.g., manual force, devices, or medications) may be employed to ensure treatment of patients at imminent risk of harm. This narrative review explores the ethical issues in restrictive practices or coercive treatment for young people with AN.

**Methods:**

345 papers were initially retrieved from online databases Ovid Medline, PsycINFO, Embase and PubMed. 247 papers were systematically screened according to established inclusion and exclusion criteria, before being assessed for relevance. 40 papers remained after screening and were included in the review.

**Conclusions:**

Only three empirical studies were identified, with the remainder of the papers in the review being non-empirical. Significant arguments supporting restrictive or coercive practices include preservation of life; cognitive immaturity; altered capacity; alleviation of pressure or guilt regarding treatment decisions (particularly for parents or guardians); and retrospective gratitude. Pertinent counterarguments include patient distress; unjust violation of autonomy; harm to others; damage to parent-child relationships; and impacts on the therapeutic alliance. Despite there being widespread agreement on the ethical issues raised, the literature lacks guidance on how to proceed in these situations. Although restrictive or coercive treatment may be considered essential, last-resort management for young people with severe AN, the literature prompts clinicians to consider the associated harms. Future empirical research should be conducted to better understand the benefits and harms of such practices from patient, parent, and clinician perspectives.

## Abstract (plain language summary)

Anorexia nervosa (AN) is an eating disorder affecting many young people. People with this disorder fear weight gain and develop unhealthy behaviours to maintain an ideal body weight and shape. With severe AN, people may need to be admitted to hospital as they can become seriously unwell from significant weight loss and malnutrition. Young people with severe AN may be treated in hospital against their wishes if there are serious concerns for their health. This may involve hospital staff using their own bodies, devices (e.g., straps) or medications to limit the person’s movement and allow treatment to occur. We reviewed all available papers discussing the advantages and disadvantages of forcing young people with AN to receive treatment in hospital. Many papers support treating people with severe AN against their wishes if their current condition is life-threatening. However, we are also prompted to consider the potential consequences of forcing treatment upon patients with AN. The review calls for further research to allow us to better understand the benefits and harms of these treatment methods from the perspectives of patients, parents, and the hospital team.

## Background

Anorexia nervosa (AN) is an eating disorder with a lifetime prevalence of 1.8% in Australia [1]and an average age of onset of 16–17 years [2]. According to the DSM-5 [3], AN is characterised by restricted energy intake resulting in low body weight (for one’s age, sex, development and physical health); an intense fear of, or persistent behaviour preventing weight gain; and altered perception of body weight and shape. The medical complications of AN typically result from severe weight loss and malnutrition [4], affecting multiple organ systems. In severe AN, hospitalisation may be required for medical stabilisation and weight gain, which can involve enteral (e.g., nasogastric tube) feeding if oral feeding is not possible [5].

Treatment refusal is not uncommon in AN, particularly due to the egosyntonic nature of the condition [6]. There are several ethical challenges that the clinical team must address when patients refuse treatment. Firstly, the team must consider the patient’s decision-making capacity and the urgency of the situation prior to using physical and/or chemical restraints [7]. Some adolescents can have decision-making capacity and may be considered mature minors. Capacity, however, can be difficult to determine in AN due to both distorted thinking and the impacts of malnutrition upon overall decision-making [8, 9]. Although it is ideal that the clinical team, young person, and family come to a mutually acceptable treatment decision, this may not always be possible. Involuntary treatment and restrictive interventions, defined as any conduct that has the purpose or effect of limiting a person’s liberty and freedom of movement, are sometimes necessary to preserve life in the treatment of severe AN [10], with one systematic review finding involuntary treatment to occur in 13–44% of AN admissions [11]. This includes involuntary hospitalisation, physical (the use by a person of their body to prevent or restrict movement), mechanical (the application of a device to restrict movement) or chemical restraint (the giving of a drug for the primary purpose of controlling the person’s behaviour by restricting their freedom of movement) [12].

This review focuses specifically on young people (under the age of 18), as AN is most prevalent in the adolescent population [2]and there are ethically relevant considerations for young people as compared to adults. Full decision-making capacity in minors requires assessment, rather than being assumed unless there is evidence of diminished capacity, as is the case for adults. In addition, there are different physiological risks and harms of malnutrition in young people. Children and adolescents may be more delayed in displaying typical signs of haemodynamic instability and left untreated, this may lead to irreversible consequences for growth and development [13, 14]. There is evidence to suggest that early and intensive measures for weight restoration may allow for catch-up linear growth and optimisation of bone density [13]. Thus, balancing harms and benefits of restrictive practices in AN in order to provide nutrition, must be performed with these considerations in mind.

This narrative review aims to assess the current literature and identify which ethical issues in restrictive practices for the treatment for AN in young people are raised, and how they are discussed. The review begins by identifying the ethical justifications given for these involuntary measures, notably the prevention of severe medical complications [15, 16], impaired capacity in AN [17], and developmental immaturity in adolescence [18]. It then addresses the ethical considerations raised against involuntary treatment and restrictive practices, such as violation of autonomy [19], and distress for the young person, family and clinicians [15, 16, 19, 20].

## Methods

### Search strategy

This narrative review involved a systematic search of online databases Ovid Medline, PsycINFO, Embase and PubMed. The complete search strategy and Boolean operators are outlined in *Supplementary Material A*.

#### Inclusion criteria

*Papers were included if they met any of the following criteria*.

I. Contained an ethical discussion.
II. Made reference to individuals younger than 18 years of age.

- Papers were retained if they mentioned both paediatric and adult patients with AN, or did not specify the age group.

III. Focused primarily on inpatient management of AN.

#### Exclusion criteria

*Papers were excluded if they met any of the following criteria*.

I. Focused on self-restraint in eating (exhibited in AN).
II. Did not include any ethical discussion.
III. Focused solely on individuals over 18 years of age.
IV. Focused solely on outpatient management of AN.

Papers were initially screened by title, abstract, then full text by the primary researcher (AY). After reviewing the inclusion and exclusion criteria, the remaining papers were assessed for relevance and any that were unclear were discussed with the broader research team. A final decision was then made regarding their inclusion in the review. All types of papers were initially considered, including those referring to both empirical and non-empirical data. Relevant papers were included if they contained explicit discussion of ethical issues using standardly recognised ethics terminology, for example, “autonomy”, “informed consent”, “capacity”, “preservation of life”, “potential of harm”, “dignity”, and “trust”. Decisions regarding what constituted standard ethics terminology were guided by LG and JO, the two clinical ethicists on the research team. Inductive content analysis was then utilised to categorise arguments based on whether they were in support of, or against the use of restrictive practices in the treatment of AN. These arguments were further sub-categorised within these two broad groups. There were no limits placed on publication dates or country of publication.

## Results

**Fig. 1:**
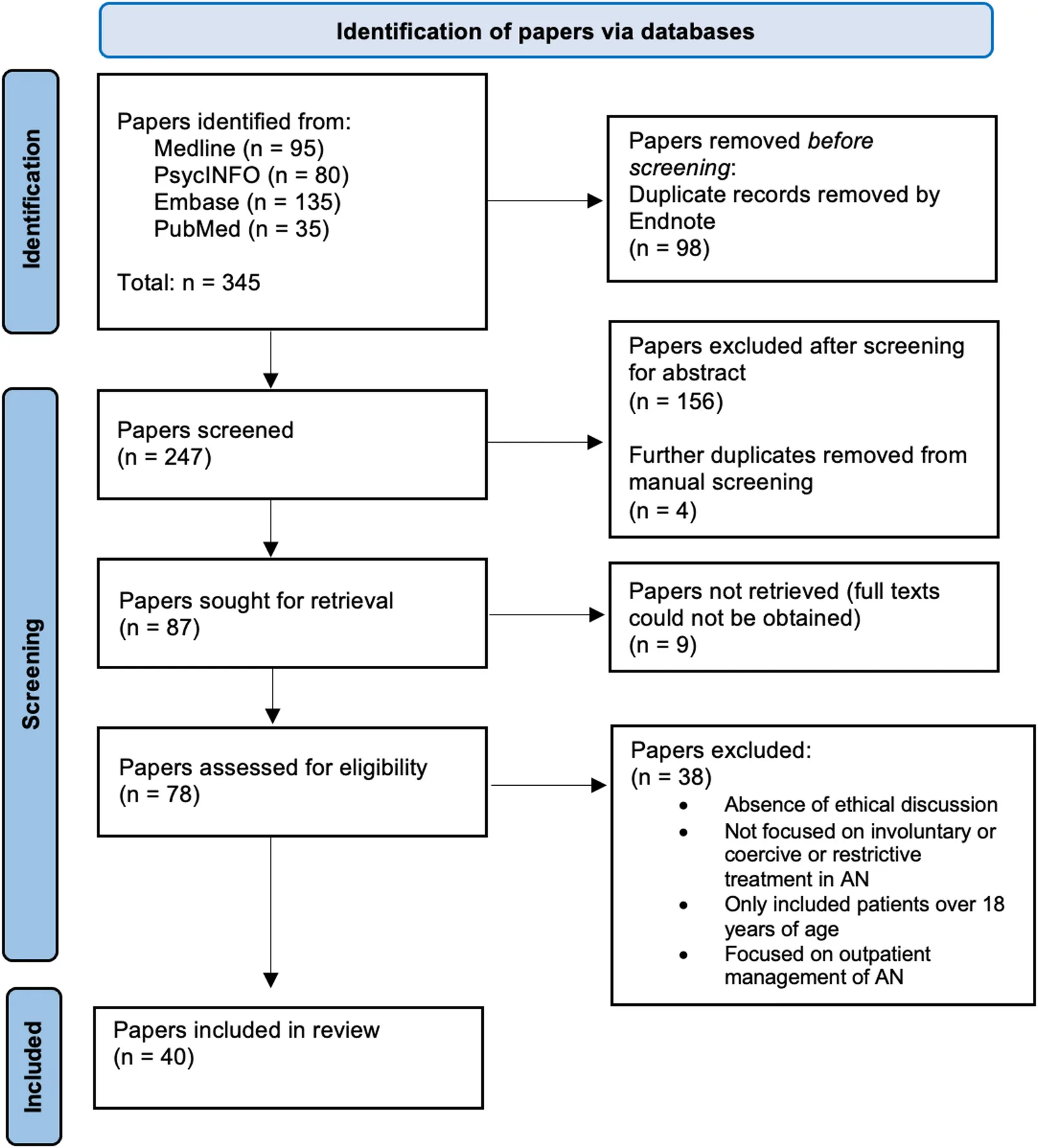
PRISMA flow diagram *(outlining number of papers at each stage of screening)*

The literature search was completed in May 2023. 345 papers were initially retrieved from the online search, with 95 from Medline, 80 from PsycINFO, 135 from Embase and 35 from PubMed. 98 duplicates were identified by Endnote 20 referencing management tool and removed, leaving 247 papers to be screened. After screening by title, abstract and full text, 40 papers remained to be included in the review. Figure 1 provides additional details regarding the number of papers at each stage of screening.

From the papers included in the review, only three were empirical studies and the remainder were non-empirical. Thirteen papers were specific to paediatric populations; ten papers referred to both paediatric and adult populations; and seventeen papers did not specify the patient age group. Thirteen papers were opinion pieces, and the remainder were primarily commentaries and review articles. Twelve papers were published in the United Kingdom, followed by ten papers published in the United States, and seven papers published in Australia. There were three from Canada; two from both Poland and Germany; and one from Denmark, Austria, Hungary, and France. While there appears to be a variety of countries represented in the literature, they are largely high-income Western countries hence potentially not reflecting the ethical values of all healthcare systems or cultures worldwide.

We identified several key themes in support of, and against the use of restrictive practices or coercive treatment in AN (outlined in Table 2). From the current literature, the key ethical themes cited in these papers in support of restrictive practices were preservation of life; the effects of developmental stage upon informed decision making; altered capacity in AN; potential alleviation of guilt; and retrospective gratitude. Conversely, the key ethical themes against the use of such practices were harm to the young person; unjust violation of patient autonomy; harm to others; and damage to relationships. The themes occurring in the papers with mixed or unspecified patient populations, were largely the same as those in the paediatric-specific papers (as demonstrated in Table 2). In particular, the papers that did not specify inclusion of paediatric patients did not raise any new issues compared to those referring to either paediatric patients only, or to both adult and paediatric patients.

**Table 1:** Summary table of papers included in the review

*Author/s*	*Title*	*Year*	Country*	*Type*	*Main issue discussed*	*Empirical or Non-empirical*	*Key ethical principles discussed*
Andersen, A.E.	Eating disorders and coercion	2007	United States	Editorial	Understanding the ethical issues associated with diagnosing and treating eating disorders and justifications for coercion.	Non-empirical	- Involuntary treatment is justified as a life-saving intervention - Retrospective gratitude
Ayton, A., Keen, C., and Lask, B.	Pros and cons of using the Mental Health Act for severe eating disorders in adolescents	2009	United Kingdom	Cohort study	Assess the advantages and disadvantages of employing the Mental Health Act (MHA) for adolescents with severe eating disorders	Empirical	- Stigma of MHA for the young person - Better long-term treatment outcomes under MHA (for patients and parents)
Bell, K.	Anorexia nervosa in adolescents: Responding using the Canadian Code of Ethics for Psychologists	2010	Canada	Commentary	Discusses ethical decision-making in the treatment of adolescents with AN (primarily for psychologists)	Non-empirical	- Importance of autonomy vs danger to patient’s health in AN treatment - Respecting patient dignity - Impaired ability to provide informed consent in AN - Different developmental stages of adolescence affecting decision-making - Retrospective gratitude
Bowers, W.A.	Civil commitment in the treatment of eating disorders and substance abuse: Empirical status and ethical considerations	2014	United States	Book chapter	Discusses ethical considerations for the use of civil commitment in treating eating disorders and substance abuse	Non-empirical	- Balancing beneficence with patient autonomy in AN treatment - Impaired capacity in eating disorders - Retrospective gratitude
Carney, T.	Anorexia: A role for law in therapy?	2009	Australia	Opinion piece	Discusses ethics issues surrounding the use of legal coercion in AN	Non-empirical	- Impaired ability to provide informed consent in AN - AN patients being unaware of the severity of their illness - Legal coercion in AN
Carney, T.	The incredible complexity of being? Degrees of influence, coercion, and control of the "autonomy" of severe and enduring anorexia nervosa patients: Commentary on "Anorexia nervosa: The diagnosis: A postmodern ethics contribution to the bioethics debate on involuntary treatment for anorexia nervosa" by Sacha Kendall	2014	Australia	Commentary	Discusses ethical issues in treating severe and enduring AN (SE-AN) and comments on arguments made by Sacha Kendall	Non-empirical	- Impaired capacity to make treatment decisions in SE-AN patients
Carney, T., Tait, D., Wakefield, A., Ingvarson, M., and Touyz, S.	Coercion in the treatment of anorexia nervosa: Clinical, ethical and legal implications	2005	Australia	Cross-sectional study	Identifies legal and ethical implications of results from a data analysis conducted from an Australian specialist AN treatment unit over almost 5 years.	Empirical	- Difficulties balancing beneficence and autonomy in coercive AN treatment - Issues providing informed consent in AN - Risk of harm to therapeutic relationship with coercion
Clausen, L.	Perspectives on Involuntary Treatment of Anorexia Nervosa	2020	Denmark	Review article	Identifies patient perspectives of involuntary treatment in AN and discuss directions for future research.	Non-empirical	- Impaired decision-making capacity in AN - AN patients’ motivation to change is shown to increase with admission (including involuntary) - Involuntary treatment may be viewed as a form of punishment in AN patients
Draper, H.	Anorexia nervosa and refusal of nasogastric treatment: A reply to Simona Giordano	2003	United Kingdom	Commentary	Responds to arguments made by Simona Giordano regarding ethics of enabling nasogastric tube refusal in AN	Non-empirical	- Difficulties balancing patient autonomy with preservation of life in AN - Challenges establishing competence in AN (especially for adolescents) - It may be justified for AN patients to refuse life-saving interventions if based on quality of life considerations
Fuller, S.J., Chapman, S., Cave, E., Druce-Perkins, J., Daniels, P., and Tan, J.	Nasogastric tube feeding under physical restraint on paediatric wards: ethical, legal and practical considerations regarding this lifesaving intervention	2023	United Kingdom	Case study and commentary	Discusses ethical, legal and practical implications of nasogastric tube feeding under physical restraint for children and adolescents admitted with AN	Non-empirical	- Nasogastric feeds can be life saving in AN - Retrospective gratitude - Forced nasogastric feeds can lead to further self-sabotaging and non-adherent behaviour - Importance of patient autonomy - Parental or guardian distress - Hospital staff distress
Giordano, S.	Anorexia nervosa and refusal of nasogastric treatment: A response to Heather Draper	2003	United Kingdom	Commentary	Responds to arguments made by Heather Draper regarding involuntary treatment for feeding purposes in AN	Non-empirical	- Complexities of determining competence in AN - Impaired ability to provide informed consent in AN - Importance of patient autonomy
Giordano, S.	Anorexia and refusal of life-saving treatment: The moral place of competence, suffering, and the family	2010	United Kingdom	Opinion piece	Discusses the circumstances under which treatment refusal should be respected in AN and compares some cases of AN to other terminal illnesses.	Non-empirical	- AN may alter patient competence to make treatment decisions - Whether to respect treatment refusal may also be determined by degree of patient suffering - May not be justified to accept treatment refusal if there is a high likelihood of recovery
Giordano, S.	Anorexia nervosa: A case for exceptionalism in ethical decision making	2019	United Kingdom	Opinion piece	Analyses philosophical and ethical issues associated with coercive treatment in AN, and provides suggestions for ethical decision making	Non-empirical	- Discusses how patient capacity is determined, and how this is impacted in AN - Also identifies flaws in the argument that AN patients lack capacity - Unpacks meaning of best interests for the patient - Retrospective gratitude
Griffiths, R.A.	Compulsory treatment and anorexia nervosa	1996	Australia	Review article	Discusses the ethical issues surrounding involuntary treatment in AN and reasons why it may be necessary at times. It also outlines various aspects of mental health legislation	Non-empirical	- Clinicians’ duty of care to protect the patient from harm - Autonomy may be breached to preserve life
Grubb, A.	Refusal of treatment and the competent patient	1994	United Kingdom	Case study	Discusses a patient’s entitlement to refuse life-saving treatment in an ethical and legal context. It refers to case studies of both adults and minors.	Non-empirical	- In contrast to adults, competent children still do not have the right to refuse life-saving interventions - The courts assume the best interests of the child - Aim to protect the minor from harm (especially irreversible physical and psychological harms) - A competent child’s wishes may be overridden where medical evidence favours treatment - Others argue the importance of valuing a competent child’s wishes in non-life-threatening conditions
Honig, P., and Bentovim, M.	Treating children with eating disorders – Ethical and legal issues	1996	United Kingdom	Review article	Discusses the ethical issues associated with involuntary treatment of children with eating disorders. It also explains applications of The Children Act (UK, 1989)	Non-empirical	- A child’s wishes should be considered in the context of age, developmental stage, and capacity - Compulsory treatment can be justified in cases where AN impairs cognitive capacity - Severe AN can have long-term consequences for physical development if untreated - Future psychological treatments for AN are unlikely to be effective until the patient reaches a safe weight
Kendall, S.	Anorexia nervosa: the diagnosis. A postmodern ethics contribution to the bioethics debate on involuntary treatment for anorexia nervosa	2014	Australia	Opinion piece	Reviews the relationship between the clinician’s power, values and perception of AN as an illness, and the ethical issues surrounding involuntary treatment in AN.	Non-empirical	- Difficulty balancing the core ethical principles (autonomy, beneficence, non-maleficence and justice) with the patient’s best interests in AN - AN may impair competence for decision-making, although competence itself is difficult to determine - Argues that involuntary treatment lacks efficacy in AN and should be a last resort - Discusses a patient’s right to refuse life-saving treatments
Kendall, S., and Hugman, R.	Social work and the ethics of involuntary treatment for anorexia nervosa: A postmodern approach	2013	Australia	Opinion piece	Discusses the ethical issues surrounding involuntary treatment in AN (primarily involuntary hospitalisation), with a focus on a social worker’s perspective. It also applies various postmodern ethics approaches to this debate.	Non-empirical	- Suggests how autonomy can be respected for patients who refuse treatment, whilst protecting them from harm - Whether involuntary treatment is justified depends on competence - Patient quality of life also considered in ethical debate - Argues that involuntary treatment lacks efficacy in AN
Lavoie, M., and Guarda, A.S.	How Should Compassion Be Expressed as a Primary Clinical and Ethical Value in Anorexia Nervosa Intervention?	2021	United States	Case report and commentary	Assess the ethics of using force in the treatment of adolescents with severe AN (in the context of a case study)	Non-empirical	- Forced interventions justified if capacity to make treatment decisions is impaired - Justified in circumstances with high risk of death or serious complications (i.e., life saving) - AN can impair capacity - Retrospective gratitude
Manley, R.S., Smye, V., and Srikameswaran, S.	Addressing complex ethical issues in the treatment of children and adolescents with eating disorders: Application of a framework for ethical decision-making	2001	Canada	Opinion piece	Provides a framework for ethical decision making that can aid clinicians in engaging adolescents with eating disorders and their families, prior to onset of an illness-related crisis.	Non-empirical	- Developmental differences in adolescents - Family involvement in decision making - Autonomy of children - Capacity and competence of a child to contribute to treatment decisions - AN can impair capacity
Matusek, J.A., and O'Dougherty Wright, M.	Ethical dilemmas in treating clients with eating disorders: A review and application of an integrative ethical decision-making model	2010	United States	Opinion piece	Outlines the ethical issues associated with treating eating disorders, then presents an ethical decision-making guide for clinicians	Non-empirical	- Clinicians’ duty of care to protect patients from harm - Patient autonomy - Debates whether compulsory treatment is a life-saving intervention - Retrospective gratitude - Damage to therapeutic alliance - Higher medical risk in children and adolescents with severe eating disorders - Developmental stage affecting treatment course - Difficulties in determining competence, capacity and cognition in those with eating disorders
Neiderman, M., Zarody, M., Tattersall, M., and Lask, B.	Enteric feeding in severe adolescent anorexia nervosa: A report of four cases	2000	United Kingdom	Case series	Discusses the ethical complexities of employing enteral feeding in young people with AN	Non-empirical	- Enteral feeding as a life-saving intervention in severe AN - AN can affect ability to make informed decisions
Radden, J.H.	Forced feeding for anorexia: Soft or hard paternalism?	2021	United States	Commentary	Responds to comments made by other authors regarding ethics of forced feeding in AN	Non-empirical	- Different developmental stages in adolescents - Potential cognitive immaturity justifying paternalism
Radden, J.H.	Food refusal, anorexia and soft paternalism: What's at stake?	2021	United States	Opinion piece	Outlines the ethical challenges associated with forced feeding in AN	Non-empirical	- Altered decision-making capacity in AN - Protecting a patient’s best interests - Forced feeding can be a life-saving intervention - Retrospective gratitude
Ramasamy, R.S.	Involuntary Treatment of Minors with Severe and Enduring Anorexia Nervosa	2021	United States	Case study and commentary	Discusses the ethical issues surrounding involuntary treatment of young people with AN (in the context of a case study)	Non-empirical	- Life-saving intervention - Involuntary treatment may lower an AN patient’s guilt for accepting nutrition - Developmental differences in adolescents - Loss of patient autonomy and dignity - Stigma associated with involuntary treatment - Impact to parent-child relationship - Distress to others (primarily parents)
Rathner, G.	A plea against compulsory treatment of anorexia nervosa patients	1998	Austria	Opinion piece	Argues against the use of compulsory treatment for AN patients	Non-empirical	- Balancing paternalism and patient autonomy - Combatting immediate danger to the patient - Lack of clear evidence proving efficacy of compulsory treatment in AN - Compulsory treatment may prompt further resistance from the patient - Difficulties with patients denying illness - Competence in patients with severe AN to make treatment decisions - Concept of control in AN patients - Retrospective gratitude
Silber, T.J.	Justified paternalism in adolescent health care. Cases of anorexia nervosa and substance abuse	1989	United States	Case study	Discusses the ethics of paternalistic approaches to treating adolescents with AN and substance abuse (describes cases)	Non-empirical	- Justified paternalism in AN - Preservation of life vs autonomy - Retrospective gratitude
Silber, T.J.	Treatment of anorexia nervosa against the patient's will: ethical considerations	2011	United States	Case study and commentary	Discusses the ethics of justified paternalism in the treatment of adolescents with AN	Non-empirical	- Patient autonomy - Impact of AN upon decision making - Retrospective gratitude
Starzomska, M.	The role of broad and narrow definitions of capacity in treating anorexic patients	2006	Poland	Review article	Explains the notion of treatment resistance and refusal and discusses the ethics of involuntary treatment in AN	Non-empirical	- Life-saving intervention - Patient autonomy - Challenges surrounding competence and capacity in AN - Compulsory treatment may increase risk of self-harm/suicide
Starzomska, M.	Ethical challenges in forcible feeding among patients with anorexia nervosa and prisoners	2006	Poland	Opinion piece	Discusses the ethical issues surrounding forced feeding of AN patients and prisoners	Non-empirical	- Protecting patients from harm - Breaching autonomy - Harms of forcible feeding - Destruction of therapeutic relationship
Tan, J.	The anorexia talking?	2003	United Kingdom	Case study and commentary	Discusses the ethics of paternalistic treatment in AN and issues surrounding competency	Non-empirical	- Protecting patients from harm - Breaching autonomy - Competency in AN
Tan, J., Hope, T., Stewart, A., and Fitzpatrick, R.	Control and compulsory treatment in anorexia nervosa: the views of patients and parents	2003	United Kingdom	Cross-sectional study	Explores the opinions of adolescents with AN and their parents, regarding competence to provide/withhold consent for treatment	Empirical	- Breaching autonomy - Difficulties surrounding capacity and competence in AN - Need for self-control and loss of control in AN - Impact of forced interventions upon future treatment decision making - Distress to AN patients and their parents - Life-saving treatment
Tan, J., Stewart, A., Fitzpatrick, R., and Hope, T.	Competence to make treatment decisions in anorexia nervosa: Thinking and processes and values	2006	United Kingdom	Commentary	Discusses capacity and competence in AN and ethics of involuntary treatment	Non-empirical	- Issues of capacity and competence in AN - Long-term efficacy of involuntary treatment
Thiels, C.	Forced treatment of patients with anorexia	2008	Germany	Review article	Considers the clinical, ethical and legal implications of forced feeding for AN patients	Non-empirical	- Life-saving intervention - Protecting patient dignity
Thiels, C., and Curtice, Jr., M.	Forced treatment of anorexic patients: part 2	2009	Germany	Review article	Update of an earlier paper considering clinical, ethical and legal implications of forced feeding in AN patients	Non-empirical	- Life-saving intervention - Involuntary interventions can reduce guilt experienced by AN patients for accepting nutrition
Touyz, S.W., and Carney, T.	Compulsory (involuntary) treatment for anorexia nervosa	2010	Australia	Book chapter	Discusses the ethical and legal considerations of coercive treatment in AN.	Non-empirical	- Life-saving interventions - Best interests of the patient vs respecting autonomy - Restoration of capacity and autonomy following intervention - Retrospective gratitude - Potential anosognosia in AN
Tsiandoulas, K., McSheffrey, G., Fleming, L., Rawal, V., Fadel, M.P., Katzman, D.K., et al.	Ethical tensions in the treatment of youth with severe anorexia nervosa	2023	Canada	Opinion piece	Explains the ethical considerations of treating young people with severe AN who are unable to make treatment decisions. Also suggests harm-reduction strategies	Non-empirical	- Life-saving interventions that also prevent impaired development during adolescence - Capacity & autonomy in severe AN - Best interests of the young AN patient - Retrospective gratitude - Potential physical & mental trauma associated with restraints - Distress for clinicians administering involuntary treatment
Tury, F., Szalai, T., and Szumska, I.	Compulsory treatment in eating disorders: Control, provocation, and the coercion paradox	2019	Hungary	Case series and commentary	Discusses the main practical and ethical considerations of compulsory treatment in AN. Presents case vignettes	Non-empirical	- Life-saving interventions - Denial of illness & lacking insight in AN - Potential damage to therapeutic relationship - The AN patient may believe they are being persecuted
Vialettes, B., Samuelian-Massat, C., Valero, R., and Beliard, S.	The refusal of treatment in anorexia nervosa, an ethical conflict with three characters: "The girl, the family and the medical profession". Discussion in a French legislative context	2006	France	Opinion piece	Discusses the ethical challenges associated with situations where young patients with AN refuse treatment (primarily within the context of the French law)	Non-empirical	- Life-saving interventions - Importance of respecting patient autonomy
Werth, Jr., J.L., Wright, K.S., Archambault, R.J., and Bardash, R.	When does the "duty to protect" apply with a client who has anorexia nervosa?	2003	United States	Opinion piece	Assess the ethical and legal implications of involuntary treatment in patients with severe AN. Also outlines some treatment guidelines	Non-empirical	- Clinicians’ duty to protect their patients - Potentially impaired judgement & capacity to make treatment decisions in AN

^*^ *Country of first author’s stated affiliation*

Table 1 aims to summarise the main issues and key ethical principles discussed in each of the papers included in the review. Further details were also included, such as the year and country of publication, along with the nature of the study (i.e., empirical or non-empirical).

**Table 2:**
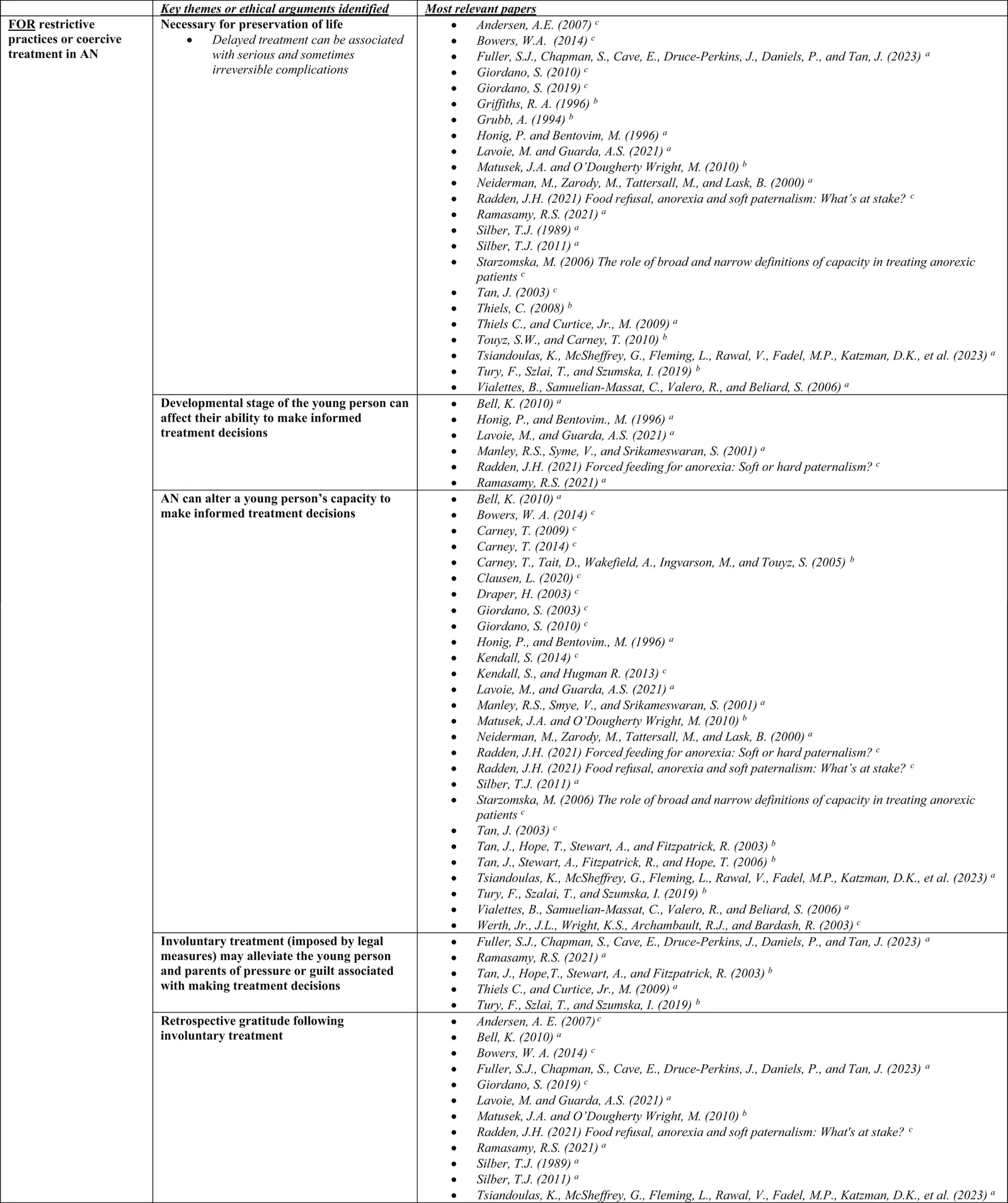

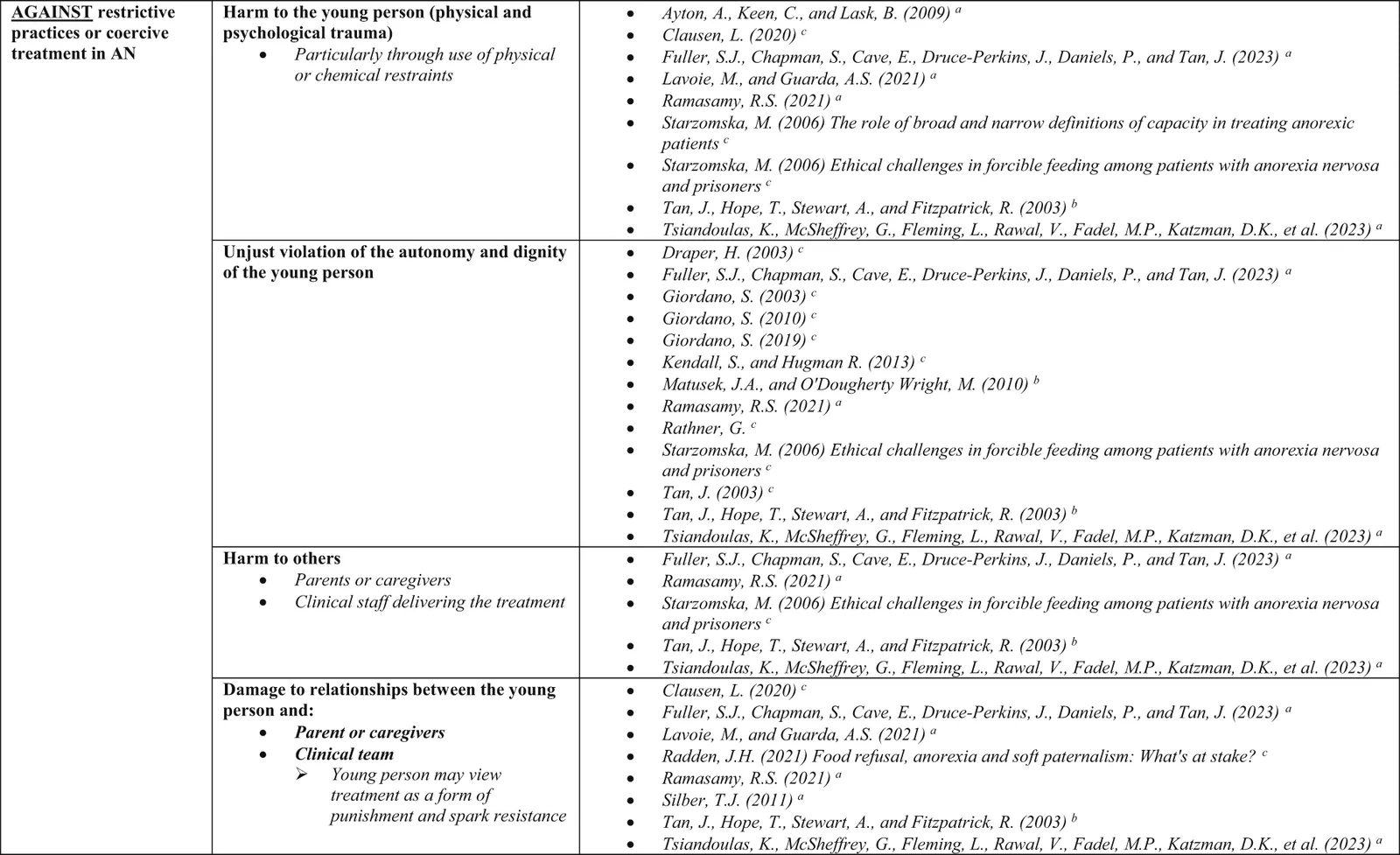
Key arguments in support of, and against restrictive practices or coercive treatment in AN

^a^ Refers to paediatric patients only

^b^ Refers to both adult and paediatric patients

^c^ Does not specify age group

Table 2 aims to summarise the key arguments in support of, and against restrictive practices or coercive treatment in AN.

## Discussion

There is much debate surrounding the ethics of employing restrictive practices for young people with AN. The following discussion will compare the arguments for and against the use of such measures, illustrating how competing ethical considerations are balanced differently in these two positions. This discussion also highlights some potential limitations of the available evidence used in these arguments. Only three empirical studies were identified through the search strategy outlined, with the remainder being non-empirical papers. Hence, the narrative review largely summarises published opinions, rather than scientific outcomes or rigorously studied questions. Furthermore, various countries are represented in the literature. Differences in healthcare systems and ethical values may have informed the commentaries provided, however given that predominantly high-income Western countries are being reflected, the papers may not account for significant societal or cultural differences between healthcare systems.

### Ethical arguments in support of restrictive interventions and coercive treatments in AN

#### Preservation of life

Several papers highlight how involuntary treatment, particularly for nutritional rehabilitation, is often crucial as a life-saving intervention in AN. This is generally reserved for cases of severe illness with high risk of mortality in young people with AN [19]. At times, clinicians must take decisive actions to prevent deterioration of their patient’s health and wellbeing. These circumstances include when the young person with AN is unable to understand the serious nature of their condition and continues to have a strong urge to maintain self-control [21]. Delayed treatment can be associated with life-threatening or irreversible medical complications such as severe malnutrition, hypotension (or cardiovascular compromise), significant hypoglycaemia or electrolyte disturbances (particularly hypokalaemia and hypophosphatemia) [15]. Failure to address severe AN in young people may also be associated with delayed or arrested pubertal growth and development, altered brain structure and function, along with reduced bone mineral density [16]. It is argued that short-term beneficence through involuntary feeding, outweighs the young person’s autonomy for treatment refusal in such cases to prevent these medical complications. Indeed, clinicians possess a duty to protect when they are aware that their patients pose a risk of imminent danger towards themselves, or others due to the AN [22–24]. Several authors claim that paternalism (overriding autonomy) is justified when there are solid prospects for recovery following the intervention [25]. The literature also presents case studies of young people with severe AN who resisted treatments as inpatients for nutritional rehabilitation [26]. In these case studies, enteric feeding led to individuals requesting oral intake sooner than expected. This supports the use of involuntary treatment and restrictive interventions as they may be necessary for a more efficient and effective recovery. The clinical team, however, should aim to restore autonomy as efficiently as possible [27–29].

#### Effect of developmental stage upon informed decision making

Several papers also comment on the developmental stage of the young person as an important consideration for using restrictive treatment in AN. Young people may lack the maturity to utilise decision-making skills needed for true autonomy. They may also vary in their abilities to understand somewhat abstract concepts including self-awareness [18], and may lack insight into the seriousness of their condition [15, 19]. In these cases, they may not be able to provide truly informed consent or refuse treatment [18]. Several authors highlight how stages of physical, cognitive and social development must be considered to enable the most appropriate approach to ethical decision-making [30]. Younger patients may be less equipped to communicate their concerns as they are accustomed to having others speak on their behalf. Young people involved in decision-making about their treatment may also feel overwhelmed and unable to speak freely due to the presence of the clinical team who they may perceive as intimidating or judgemental [30]. Hence, cognitive immaturity is considered by some to be a justification for paternalism in paediatric populations [31].

#### Altered capacity in AN

Furthermore, altered capacity from AN can impair young people’s ability to make informed decisions about their care. Individuals are deemed competent to make medical decisions when they can understand the nature of the treatment and be aware of its purpose. They must also be able to consider the associated risks and benefits [32]. The reviewed literature introduces the idea that severe AN impairs capacity [17], which may prevent the young person from being able to make autonomous decisions [27–29]. Individuals with eating disorders do not realistically perceive true signals of appetite and satiety, making it difficult to determine when and how much to eat at times [32]. In severe AN, competence levels can be altered early in the starvation process [18]. Some young people with AN may deny their illness altogether, rendering any form of treatment involuntary and forced in their view [33, 34]. It is also argued that although individuals with AN are often clear that they do not want to die, they may not perceive their risk of death to be imminent [12, 35]. Such individuals are not considered to have full capacity to refuse treatment and involuntary treatment may be justified.

#### Potential alleviation of guilt

In addition, involuntary treatment imposed by legal measures, may alleviate individuals (young people, parents, or guardians) of the pressure and guilt associated with making treatment decisions in severe AN. Parents may feel pressured by their child to withhold consent for treatment. This can result in them allowing their child to be prematurely discharged from hospital, or they may continue to tolerate disordered eating behaviours at home [19]. Involuntary treatment may reduce the young person’s feelings of guilt and self-blame for accepting further nutrition and may also have the effect of allowing the young person to understand the true seriousness of their clinical state [19]. Given the intrusiveness of forced feeding that directly conflicts with the young person’s wishes and causes significant distress, some also consider it unjust to ask parents to make choices that may damage their long-term relationship with their child [20]. Being free of the responsibility to make treatment decisions, young people and their families may focus on their shared goal of recovery.

#### Retrospective gratitude

Finally, several papers highlight that following inpatient treatment for AN, many young people express retrospective gratitude for coercive measures employed. During the recovery phase, young patients and their caregivers often appreciate earlier compulsory interventions, despite previous objections, if they understand that such treatments were done in their best interests to preserve life [15, 17, 18, 20]. This is particularly pertinent in cases where young people have solid relationships with their caregivers and treating team [19]. The literature also identifies that many young people with AN and their parents do not support clinicians allowing individuals to die by accepting their refusal of treatment [36]. Although retrospective gratitude is not usually a justification for paternalism in medicine, there is an argument that AN presents an exceptional circumstance where this is sometimes considered [25].

### Ethical arguments against the use of restrictive interventions and coercive treatments in AN

#### Harm to the young person

In contrast to the arguments discussed above, many papers highlight that restrictive or coercive practices, particularly the use of physical and chemical restraints, can be distressing to the patient. One paper outlines a case of a 17-year-old female patient with severe AN who continued to pull out her nasogastric tube (NGT), resulting in several re-insertions [15]. Repeated NGT re-insertions along with the use of chemical restraints, may cause the patient psychological and physical trauma [15, 16]. Physical consequences include trauma to the nasopharynx, aspiration pneumonia and oesophageal perforation. Regarding psychological trauma, some authors suggest that disordered eating behaviours may provide some young people with AN with a sense of security or control [16]. Hence by treating their condition, these individuals may feel as though a part of their identity is being removed. Such consequences should be considered when deciding to employ restraint under involuntary treatment.

#### Unjust violation of patient autonomy

Several authors also consider involuntary treatment in AN to be an unjustified violation of patient autonomy. The principle of autonomy promotes an individual’s entitlement to informed choices about their care, without coercion or external influences [22]. In general, clinicians should respect their patients’ decisions, irrespective of their own personal opinions. However, ethical issues arise under circumstances where the individual is deemed incompetent to make certain treatment choices [16, 22, 32, 37, 38]. Young people and their families may feel that their dignity and autonomy have not been respected when restrictive measures are employed. Involuntary treatment is also seen by some as a cause of psychosocial harm to patients and may be considered stigmatising for the young person with AN [19]. A limited number of papers raise the idea that a small minority of individuals with long-term severe AN may be entitled to refuse life-saving interventions [32], particularly if the decision is related to their quality of life (QOL) [38]. The rationale is that although individuals with AN may be deemed incompetent to make decisions regarding nutrition, they may still be competent to determine QOL in the setting of their illness [38], particularly where improvement in their clinical status is deemed unlikely.

#### Harm to others

Involuntary treatment with the use of physical and chemical restraints can also cause significant harm to others, notably the young person’s family and hospital staff assisting with these interventions. Clinical staff carrying out involuntary treatment (especially physical restraint) may experience moral distress, exhaustion, injuries, and aggression [16, 20]. Physical injuries can range from back pain and bruises, to dislocated shoulders and severe head injuries. Moral distress may be more profound with sustained interventions as clinicians may feel that their efforts are no longer aligned with their aims of providing patient-centred care [16]. Staff may also experience negative emotions (e.g., anxiety or fear) and emotional or moral distress resulting from internal conflicts [16]. This is particularly difficult if staff have built a strong therapeutic relationship with the young person over time. Similarly, parents of young people living with severe, chronic or treatment-refractory AN may suffer from physical and emotional exhaustion, in addition to other psychosocial problems [19]. They may experience a range of negative emotions, from distress and helplessness to anger and frustration in circumstances where coercive treatment is needed. While parents may understand the immediate necessity of nutritional rehabilitation and be fearful for their child’s life, they may concurrently be horrified or distressed by the feeding methods employed [20].

#### Damage to relationships

On a similar note, involuntary treatment may damage the relationship between the person with AN and their parental figures, in addition to the therapeutic alliance with the clinical team. The parent-child relationship can be hindered if parental consent to treatment overrides the young person’s refusal of treatment [19]. Several papers also raise the idea that although young people with AN may understand that involuntary interventions can be lifesaving, they may also view this as a form of punishment [37, 39]. This may lead to associated psychological trauma and even increase their rebellion towards treatment. Forced feeding may spark such a large degree of resistance, that the individual flees the healthcare setting, or acquires further unhelpful behaviours towards their care [17]. Some authors explain that a young person’s sense of perceived coercion regarding treatment is correlated with feelings of not being heard by their treating team [15]. It is reiterated in the literature that early in the treatment process, it is important to build rapport with the young person, display empathetic listening and involve the young person in decisions about their care, as appropriate for their age [16]. A positive relationship between young people, family, and the healthcare team, provides a foundation for sound decision-making in future [29].

#### Limitations of the review

The screening process included relevant papers with unspecified age groups, along with those that refer to both adult and paediatric populations. Although many of the principles discussed are applicable to young people, some ethical issues of involuntary treatment in those under 18 years of age, may differ to those encountered by adults. To improve the quality of the review, the inclusion criteria could be further refined to include texts solely referring to young people under the age of 18 with AN. However, the ability to do so is limited by the lack of relevant papers focusing solely on paediatric patients. Furthermore, it is important to consider the probability that some potentially relevant papers were not identified with this standard search strategy. If the title, abstract or keywords section of a paper did not contain any ethical terminology, then this would not have been detected in the search method.

#### Limitations of available literature

Many of the papers identified, discuss the ethical issues of involuntary treatment in AN broadly, rather than unpacking the frequently used restrictive practices in greater detail. Further research is warranted to better understand the harms and benefits of commonly used interventions for feeding, such as physical and chemical restraints for NGT feeds. Furthermore, all but three of the papers included were non-empirical and constituted primarily opinion pieces, commentaries, or review articles. Hence, this narrative review reveals an overall lack of empirical research assessing the ethics of restrictive practices specifically for young people with AN. Due to the limited number of empirical papers, a narrative review rather than systematic review, was deemed the most appropriate method of reporting findings from the current literature.

#### Implications for clinical practice

The narrative review largely reveals the acceptability and utility of employing restrictive practices as a last-resort measure [20, 27] for the treatment of young people with severe AN. However, it also prompts clinicians to consider the harms associated with such practices. The literature lacks provision of an ethical framework to assist clinicians in navigating these complex situations. For example, it does not consistently use or articulate a framework for understanding autonomy or agency for individuals whose decision-making capacity is compromised in some form; or for balancing different types of potential harms against each other. This is significant because although there is agreement on the ethical issues raised, there is disagreement on the preferred course of action, with no means of resolving it.

One partial exception to this is that various papers provide suggestions to minimise the harms associated with involuntary interventions. Firstly, the clinicians can promote autonomy by offering the young person choices wherever possible [16], such as asking for preferred timing of treatments. They should also support young people in decisions surrounding oral intake to potentially avoid forced feeding altogether [20]. This may enable young people to feel more in control, allowing them to diffuse any potential anger previously directed towards their treating team [36]. Furthermore, it is important to respect a young person’s dignity, such as providing them with sufficient warning before a feed [18, 20]. There should also be an adequate number of skilled staff to carry out involuntary feeding interventions in a safe and compassionate manner [20]. Overall, minimal coercion should be utilised, and clinicians should motivate young people to eat with greater independence over time [40, 41].

#### Conclusion and implications for future research

Although involuntary treatment and restrictive practices may be considered necessary for inpatient management of young people with severe AN, the current literature largely emphasises that this should be a last-resort measure and clinicians are encouraged to consider the associated harms. Much of the literature identifies the pertinent ethical issues related to these involuntary practices but fails to provide an ethical framework to guide clinical decision-making. This review builds on the current body of literature by highlighting that there is a lack of empirical evidence to assist clinicians in assessing relative benefits and harms of restrictive practices. Furthermore, many of the papers identified are not specific to paediatric AN populations. This review calls for future research assessing the ethics of restrictive practices for the treatment of AN in young people under 18 years in a more nuanced fashion. Empirical research should be conducted to gain a deeper understanding of young person, parent, and clinician experiences in relation to specific types of involuntary treatments (e.g., restraint in feeding). Future studies may involve semi-structured interviews conducted with larger sample sizes and participants selected from more diverse inpatient institutions (including different countries).

## Data Availability

No datasets were generated or analysed during the current study.

## Abbreviations

DSM-5: Diagnostic and Statistical Manual of Mental Disorders, Fifth Edition
AN: Anorexia Nervosa
QOL: Quality of Life
NGT: Nasogastric tube
